# Opportunities to More Comprehensively Assess Sexual Violence Experience in Veterans Health Administration Medical Records Data

**DOI:** 10.1007/s11606-022-07581-7

**Published:** 2022-08-30

**Authors:** Brittany F. Hollis, Nadejda Kim, Ada Youk, Melissa E. Dichter

**Affiliations:** 1 St. Lawrence University, 23 Romoda Dr, Canton, NY 13617 USA; 2 Center for Health Equity Research and Promotion, VA Pittsburgh Healthcare System, Pittsburgh, PA USA; 3 Department of Biostatistics, University of Pittsburgh Graduate School of Public Health, Pittsburgh, PA USA; 4 Center for Health Equity Research and Promotion, Corporal Michael J. Crescenz VA Medical Center, Philadelphia, PA USA; 5 Temple University School of Social Work, Philadelphia, PA USA

## Abstract

**Introduction:**

Experience of sexual violence (SV) is prevalent among the Veteran population and associated with many negative mental and physical health outcomes including suicidal behavior, obesity, post-traumatic stress disorder, anxiety, depression, and poor sexual and reproductive functioning. Although Veterans of any gender may experience SV, women Veterans are particularly at risk. Research on SV among Veterans has focused primarily on the experience of SV during military service (military sexual trauma, MST), although Veterans may also experience SV prior to and following military service. The aim of the current study was to construct a more comprehensive method of identifying SV among Veterans Health Administration (VHA) patients as documented in medical records in a national cohort of 325,907 Veterans who used VHA care between 2000 and 2018 in order to inform future research in this area.

**Method:**

We used three indicators to identify SV in VHA medical records: (a) the MST screen, (b) the sexual violence item of the intimate partner violence (IPV) screen, and (c) International Classification of Disorders (ICD) codes (versions 9 and 10) representing adult sexual abuse and assault. Univariate descriptive analyses were conducted to determine the exclusivity and overlap of the SV measures.

**Results:**

The universal MST screen was the most commonly identified indicator of SV in the data. However, including the IPV and ICD indicators identified an additional 5% of Veterans who had experienced SV, accounting for thousands of patients.

**Discussion:**

The results of the current study indicate that using the three-pronged approach of SV collection is a more comprehensive method of identifying patient SV experience through VHA medical records and contributes uniquely to the methodology of studying social factors’ impact on health care. Clinical screening and documentation of SV allow for the assessment of health impacts and trends through examination of medical records data.

**Supplementary Information:**

The online version contains supplementary material available at 10.1007/s11606-022-07581-7.

## INTRODUCTION

Sexual violence (SV) is a serious public health concern that is associated with many negative health outcomes. The Centers for Disease Control and Prevention (CDC) defines SV as any sexual activity that occurs when consent is not given or obtained freely.^1^ SV is especially problematic for women and girls, with one in three experiencing SV during their lifetime.^1^ Additionally, rates of SV within the military may be higher than those within the general population^2,3^ with some studies finding 64% of women who have served in the military reporting a lifetime experience of SV.^4–9^ Experiencing SV can lead to acute and chronic negative mental and physical health outcomes. Women Veterans who have experienced SV have an increased suicide risk, and higher rates of obesity, post-traumatic stress disorder, anxiety, depression, and poor sexual and reproductive functioning compared to women who have not experienced SV.^7,10–14^ It is important to note that, although women Veterans are more likely to experience SV,^15^ SV is also problematic for Veteran men.^16^

There is a burgeoning field of research examining the impact of social factors on health (e.g., socioeconomic status, education, etc.),^17–19^ which has been correlated with an increase in health-care systems screening and documentation of such social factors. Of particular importance is the impact of interpersonal violence, especially SV, as a key social health factor associated with a plethora of adverse outcomes. The Veterans Health Administration (VHA) has been a leader and early adopter of the integration of social health factors screening in the electronic health record, including military sexual trauma (MST), and recently with the addition of the intimate partner violence (IPV) screen. The integration of such health factors not only is important to flag patients who may be in need of assistance due to the negative effects of such experiences, but also indicates that health records are an efficient and comprehensive means for useful health services research.

Over the past two decades, research on SV among military members and Veterans has heavily focused on the experience of MST,^4,7,10,20–24^ defined as sexual harassment or assault that occurs while an individual is in the military, regardless of relationship to the perpetrator.^10,25^ Although prevalence rates vary for men and women depending on MST measurement, approximately 1–4% of male and between 16 and 40% of female Veterans report MST.^3,10,21,26^ Since 2000, the VHA has implemented a universal MST screen to be administered to all Veteran patients.^10^ The addition of this screen, and documentation in the medical record, has facilitated the examination of MST exposure in health services research that relies on medical records data.^10,21,25,27^ Documentation within the Veteran’s medical record has been beneficial in tracking MST exposure, trends, health associations, health-care utilization, and disparities. The MST screen is also the most common method of SV assessment within the Veteran population.^20,26^ Although MST research is important, as it is limited to SV occurring during active-duty military service, it does not capture pre- and post-service SV experience, which may also be prevalent among this population. For example, Dichter and colleagues (2015) found high rates of intimate partner violence (IPV) exposure following separation from military service, with 20% of female Veterans in this sample experiencing sexual IPV following separation from military service.^28^ While there is substantial overlap between MST and SV experience pre- and post-military service, examining MST as a singular indicator of SV potentially misses a large portion of Veterans who have non-MST SV experience outside of the military, as well as experiences of SV that may have occurred in addition to MST.

In addition to the MST screen, the VHA has more recently implemented an annual screen for IPV (either among only female patients or all patients, depending on site).^29–32^ The IPV measurement tool is a modified version of the 5-item screen (Extended Hurt Insult Threaten Scream; E-HITS) that assesses if a partner hurt, insulted, threatened, screamed, and/or forced sexual activities within the last year.^33,34^ The sexual violence item of the IPV screening allows for identification of past-year sexual violence that might have occurred outside of military service and, thus, might not have been captured on the MST screen. However, the screen does not include any SV experiences that occur external to a current or previous intimate relationship. While the MST and IPV screening tools are useful for identifying some SV experiences, they do not capture SV that occurs outside of military service or in non-IPV situations (i.e., non-partner sexual violence), even though these SV experiences may be disclosed and recorded as part of the health-care visit (i.e., reported as a diagnostic code in the electronic health record).

A third way to identify SV in medical records is through medical records codes (International Classification of Disorders [ICD] codes 9^th^ and 10^th^ edition) related to sexual assault or abuse in childhood or adulthood. Although prior research has found that ICD codes for abuse in adults are inconsistently and infrequently used in medical records documentation,^35–39^ when they are applied, they may capture additional SV experience that do not necessarily occur while in the military and/or is not perpetrated by an intimate partner. An example may be a veteran patient who comes to the ER following a non-partner sexual assault—this incident may be captured in an ICD code but would not be captured through an IPV or MST screen.

Experiences of SV outside of MST or sexual IPV (e.g., non-partner adulthood sexual violence outside of military service and childhood sexual violence) may impact Veteran health and health care and may be missed by research relying on MST or IPV screens. In the current study, we sought to examine a more comprehensive method of identification of SV within the VHA medical records using a three-indicator (three-pronged) approach. Three indicators were used to identify SV documentation in the medical record: (1) MST screen, (2) SV item of the IPV screen, and (3) ICD codes for SV in childhood and adulthood. As this approach has not previously been examined in research, we also report trends over time for each indicator. This project is unique as it is not only seeking to find a more comprehensive measure of SV within VHA medical records data, but also includes men.^4,20,32,40^ Due to the negative impact of SV on health, it is vital to study the impact and trends of SV among both women and men, while expanding the measurement of SV in order to broaden research findings and shape appropriate VHA treatments.

## MATERIALS AND METHODS

### Data Sample

Data were extracted from the VHA Corporate Data Warehouse (CDW), a national repository of VHA electronic medical records data. The cohort for the study included 325,907 Veterans with any documented SV indicator in VHA medical records between 2000 and 2018. Since 2000, all VA medical facilities have implemented a universal one-time MST screening.^41,42^ As of 2018, 39 of 146 VHA facilities had initiated routine IPV screening with sites spread out across 28 states (and the District of Columbia). Additionally, all VA health-care providers may use ICD codes to classify patient symptomology; however, it is unknown how regularly providers indicate these codes in the patient’s medical record. Approval for the study was granted by the Pittsburgh VA Medical Center Institutional Review Board.

### Measures

Both MST and IPV screening within the VA are conducted by a health-care provider, such as a nurse, health tech, or primary or specialty care provider, prompted by a “clinical reminder” embedded within the electronic health record (EHR). The MST screen is assigned to be conducted only once within a lifetime given that it assesses for a historical event prior to engaging in VHA care. The IPV screen assesses for past-year IPV experience and thus may be repeated over time. ICD codes can be recorded at any point in the course of a Veteran’s care. In the current study, clinical reminder responses were collected for the MST and IPV screens, and ICD codes were pulled, for fiscal years 2000–2018 among all patients who had a visit in 2018.

The VA uses a 2-item screen to assess for MST: “While you were in the military, a) Did you receive uninvited and unwanted sexual attention, such as touching, cornering, pressure for sexual favors, or verbal remarks? b) Did someone ever use force or threat of force to have sexual contact with you against your will?”^42^ An affirmative response to either of those questions is considered MST positive.

A modified version of the Extended Hurt Insult Threaten Scream (E-HITS) is used as the screening tool for intimate partner violence.^32,33^ It is a five-item measure that asks individuals how often in the past year (from 1 = *never* to 5 = *frequently*) a current or former partner: physically hurt you, insulted or talked down to you, threatened you with harm, screamed or cursed at you, or forced you to have sexual activities. The current study used the forced sexual activities question to determine sexual IPV. A score greater than 1 on the forced sexual activities item was an indicator of a positive response to having experienced SV from an intimate partner. MST and IPV screen responses are recorded in the VHA medical record as coded fields (“health factors”).

ICD codes are health-care classifications for patient symptomology or experience noted in the medical record by the provider. The current study used all ICD 9^th^ and 10^th^ edition codes related to SV (i.e., childhood and adulthood sexual abuse and assault; for the list of codes, see Supplementary Appendix). The VHA moved from ICD 9^th^ to 10^th^ edition codes in 2015. ICD codes for sexual violence may be applied in conjunction with or independent of a positive MST or IPV screen. The ICD codes may also capture disclosures of sexual violence that do not occur through screening and/or do not fit within the operational definitions of MST or IPV.

### Statistical Analysis

The first step was to identify those individuals who had documentation of a MST or sexual IPV health factor, or ICD code related to SV (denominators or the total number of people screened/reporting ICD codes is available; see Supplementary Appendix). Once the cohort was created, we examined frequency trends for each of these SV indicators, overall and separately for male and female patients. Secondly, descriptive statistics for the SV measures (i.e., frequencies and percentages) were examined among the overall sample, as well as stratified among males and females.

Due to variation in the initiation of screening and changes in ICD coding, we examined data in waves. The VHA began national MST screening in 2001. As this screen is designed to be administered once in a lifetime (given that it is administered primarily to those no longer serving regarding past experience during military service), we expected the initial year of implementation to capture the most Veterans (and thus the greatest number of positive screens), followed by a plateauing in subsequent years as only Veterans newly entering the system or otherwise not previously screened would be included. The IPV screen was initiated in 2014 with gradual rollout across VHA sites; we thus expect to see positive IPV screens to first appear in 2014 with increases due to uptake across sites in subsequent years, with most sites implementing the screen in 2016. In order to detect trends, the data were examined in two waves: the first from 2000 to 2015, and then 2016–2018. Analyses were conducted on a case level, not patient level, in order to determine overlap between the SV variables. All analyses were conducted using Stata.^43^

## RESULTS

### Trends Over Time

**Figure 1:**
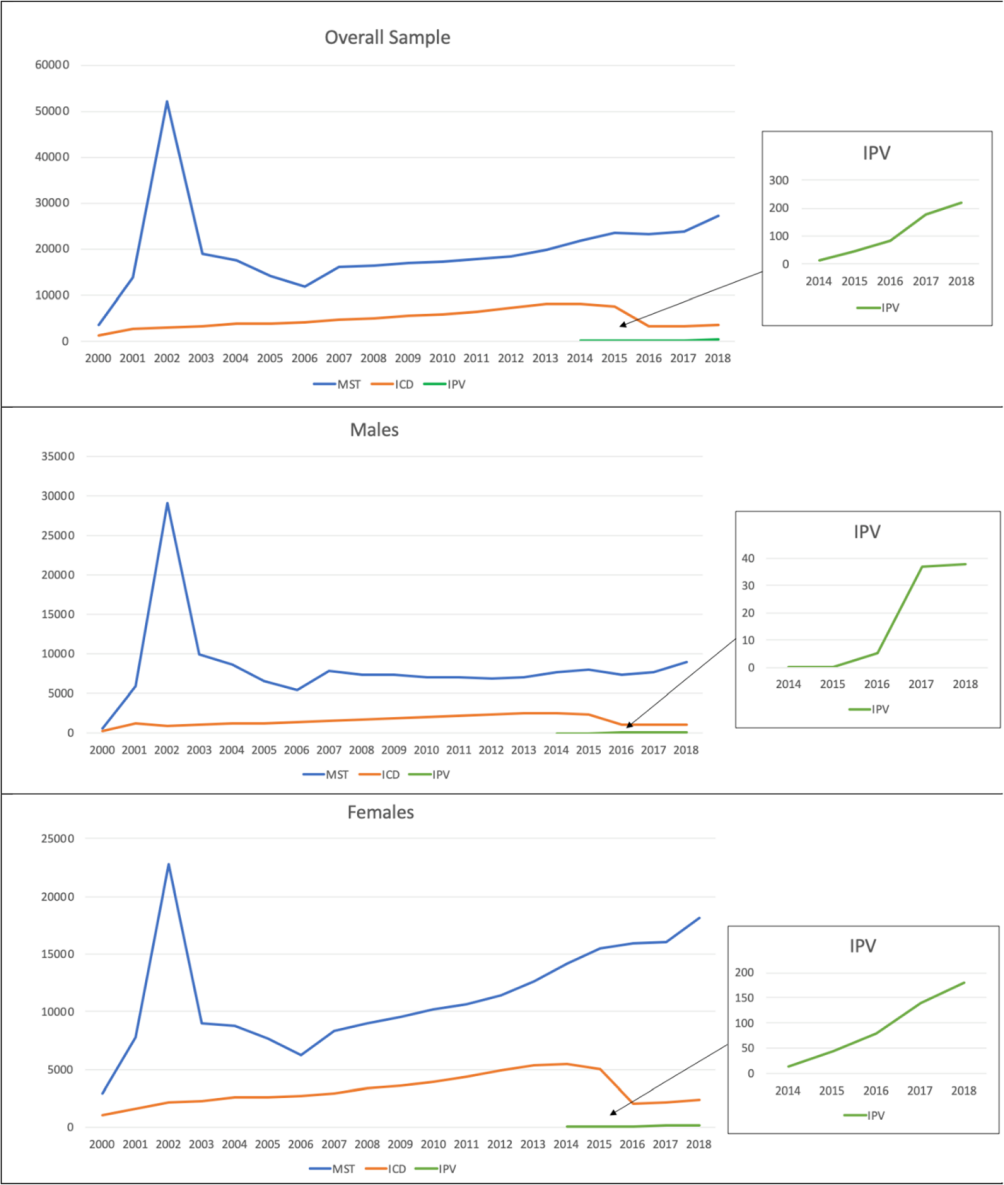
Screening and ICD code trends from 2000 to 2018 for males, females, and the overall sample.

Our cohort included 325,907 patient records, 145,547 (44.7%) male and 183,360 (56.3%) female Veterans (cases *N* = 328,292) who screened positive for either MST or partner SV, and/or had sexual abuse or assault ICD codes from 2000 to 2018. As indicated in Figure 1, trends for the overall sample indicate that the number of positive MST screens peaked in 2002, with a decline then until 2006 and a relatively steady increase between 2006 and 2018. Reports of ICD codes for SV peaked from 2013 to 2014 followed by a sharp decline and a recent small increase. Since implementation of the IPV screen in 2014, rates of partner SV report have risen exponentially.

Descriptive analyses were conducted among the overall sample, as well as stratified among male and female patients, in two waves (2000–2015 [wave I] and 2016–2018 [wave II]). Results below are separated by waves to assess the overall sample and then broken down by gender. For visual representations, see Tables 1 and 2.

**Table 1:** Screening and ICD Code Percentages for Wave I (2000–2015) in Males, Females, and the Overall Sample

Variable	*n*	% of total	*n*	% of total	*n*	% of total
	Overall sample		Males		Females	
ICD only	8211	3.1%	5096	4.1%	3114	2.2%
MST only	224,107	85.7%	108,550	88.4%	115,549	83.4%
IPV only	36	0.0%	–	0.0%	36	0.0%
ICD + MST	29,058	11.1%	9178	7.5%	19,878	14.3%
ICD + IPV	–	0.0%	–	0.0%	–	0.0%
MST + IPV	17	0.0%	–	0.0%	17	0.0%
ICD + MST + IPV	4	0.0%	–	0.0%	4	0.0%
**Total**	**261,433**	100.0%	**122,824**	100.0%	**138,598**	100.0%
Any (total) ICD	37,273	14.3%	14,274	11.6%	22,996	16.6%
Any (total) MST	253,186	96.8%	117,728	95.9%	135,448	97.7%
Any (total) IPV	57	0.0%	–	0.0%	57	0.0%
ICD with MST	29,062		9178		19,882	
ICD with IPV	4		–		4	
MST with IPV	21		–		21

**Table 2:** Screening and ICD Code Percentages for Wave II (2016–2018) in Males, Females, and the Overall Sample

Variable	*n*	% of total	*n*	% of total	*n*	% of total
	Overall sample		Males		Females	
ICD only	2938	4.4%	1048	4.7%	1890	4.2%
MST only	61,074	91.3%	20,473	91.7%	40,593	91.2%
IPV only	363	0.5%	71	0.3%	292	0.7%
ICD + MST	2390	3.6%	737	3.3%	1653	3.7%
ICD + IPV	15	0.0%	1	0.0%	14	0.0%
MST + IPV	72	0.1%	7	0.0%	64	0.1%
ICD + MST + IPV	7	0.0%	–	0.0%	7	0.0%
**Total**	**66,859**	100.0%	**22,337**	100.0%	**44,513**	100.0%
Any (total) ICD	5350	8.0%	1786	8.0%	3564	8.0%
Any (total) MST	63,543	95.0%	21,217	95.0%	42,317	95.1%
Any (total) IPV	457	0.7%	79	0.4%	377	0.8%
ICD with MST	2397	–	737	–	1660	–
ICD with IPV	22	–	1	–	21	–
MST with IPV	79	–	7	–	71	–

### SV Measurement Overlap

**Figure 2:**
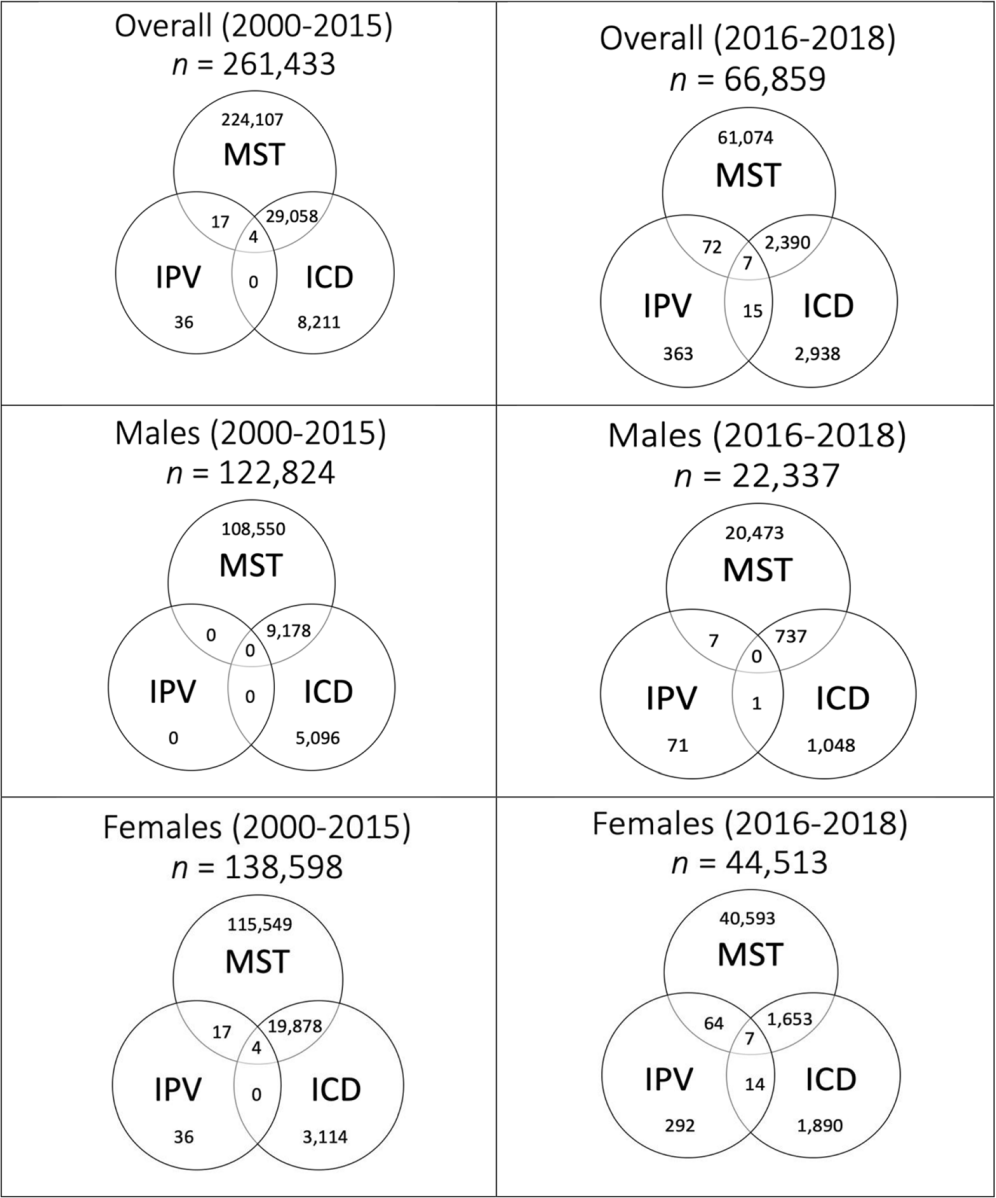
Overlap numbers and percentages in screening and ICD codes in wave I and II for males, females, and the overall sample.

Figure 2 demonstrates the overlap in the three indicators (positive MST screen, positive sexual IPV screen, ICD code for SV) among the sample, separately for each wave and by overall, male, and female. In wave I (2000–2015), the majority of Veterans with SV documentation (96.8%) had documentation of a positive MST screen; 3.2% (8247) of those with SV documentation were identified independent of the MST screen. The inclusion of IPV and ICD indicators of sexual violence identified an additional 3316 (5%) across 2016–2018 that would not have been identified through assessment of the MST variable alone.

## DISCUSSION

Results indicate that, for research, evaluation, and quality improvement efforts interested in sexual violence not limited to MST, using the MST and IPV screening, as well as ICD codes, allows for a more comprehensive approach to the identification of SV compared to only one or two of the indicators. Expanding the number of Veterans identified as having SV can help researchers to broaden the understanding of SV and help target appropriate treatment. Limiting SV assessment to MST screen indicators alone excludes approximately 5% of all documented cases of SV within VHA records. Adoption of IPV screening that includes an SV measure provides additional information on Veterans’ IPV experiences.

Electronic health records research does not require any recruitment or primary data collection allowing for more researchers to have access to VA patient outcomes, with the caveat that data is limited to what is maintained in the health record. The results of the current study suggest that using the three-pronged method of SV collection is a comprehensive method of gathering SV data among VHA patients and contributes uniquely to the methodology of studying social factors’ impact on health care specifically among female Veterans.

### Limitations

Although this study is unique and contributes to the research on SV measurement among female Veterans, there are a few limitations. First, this method of SV collection is dependent on patient reports and health-care provider documentation of SV experience, missing undisclosed/documented cases. SV is one of the most underreported of all experiences, which means the numbers in this study are likely an underrepresentation of the actual number of SV cases among this population.^44^ Yet, research suggests that patients want to be screened for SV and are highly likely to report upon screening.^45–47^ Secondly, the MST screen is a universal screen that has been in implementation for two decades. However, implementation of the IPV screen is newer and, in most cases, limited to women patients. Future research should continue to monitor its implementation and use in SV measurement. Third, although clinicians are able to use ICD codes to denote specific patient condition or experience, it is unknown how regularly these codes are used for SV. Future research could supplement analyses of ICD codes with natural language processing and chart review (which are far more extensive than the scope of this current analysis). Additionally, there was an update to the ICD codes; providers switched from ICD 9 to ICD 10 codes in 2015, which may account for the sharp decrease in ICD-positive codes after 2014. The long-term effects of this change on SV data are unknown, and future research should continue to collect and examine ICD code data. Lastly, as this study was a retrospective data analysis, the researchers were unable to examine the number of patients who declined to answer the screening questions or who were not screened at all.

### Implications

The practical implications of the findings of the current study highlight the more comprehensive identification of SV cases when using the three-pronged approach compared to only one or two of the indicators. Although there was overlap between the three prongs, each contributed unique data points. Meaning, each prong was responsible for contributing a certain percentage of unique sample data per respective method of collection (i.e., the number of individuals with sexual IPV who were only captured with the IPV screen). This three-pronged method of SV measurement helps to break through the silos of screening and ICD codes to include individuals who would have otherwise been omitted.

### Conclusions

It is vital to continue to expand research on SV among female Veterans beyond MST, as there are many negative health consequences as a result of SV experienced in partner relationships,^48–50^ as well as childhood and adulthood sexual abuse/assault.^51–53^ Furthermore, SV survivors often experience more than one form of sexual abuse/assault,^54,55^ and the effects of such cumulative trauma can have a negative impact on an individual’s health.^56,57^ Research with women veterans has identified multiple time periods of sexual violence experience and association with increased risk over time, highlighting the need for such more inclusive measures of sexual violence.^58^ Therefore, it is imperative for future research to expand the methods in which SV is studied in the VA women Veteran population as research inherently impacts treatment. Additionally, future research could take this method of SV assessment and use it to examine potential impacts on patient outcomes.

Previous research has focused largely on MST as an indicator of SV among female Veterans. While it is important to continue research on the effects of MST, SV also takes place outside of the military experience, such as SV that occurs prior to or after military service. Research that focuses strictly on MST may not account for these types of SV experiences. The current study found support for a three-pronged approach to SV measurement within VA data. This approach entailed the use of the MST and IPV screen, along with ICD codes relevant to sexual abuse and assault. It was found that this three-pronged approach contributed unique data points including thousands of women who would have otherwise been omitted if this study focused strictly on the MST screen. The findings of this study encourage future research to use this approach in order to expand knowledge on the effects of SV among the women Veteran population in an effort to help reduce disparities and improve care at the organizational level.

## Supplementary Information

ESM 1 (XLSX 12 kb)

ESM 2 (DOCX 14 kb)

## Declarations

### Disclaimer

The opinions expressed in this work are the authors’ and do not reflect those of the Department of Veterans Affairs or the U.S. government.
