# Supplementary material for: Opportunities to More Comprehensively Assess Sexual Violence Experience in Veterans Health Administration Medical Records Data
Source: J Gen Intern Med. 2022 Aug 30;37(Suppl 3):734–41. doi: 10.1007/s11606-022-07581-7 (PMC9481829; doi:10.1007/s11606-022-07581-7)
Supplement: Supplementary file 2 — (DOCX 14 kb) [file 11606_2022_7581_MOESM2_ESM.docx]

| Sexual Violence Codes | |  |  |
| --- | --- | --- | --- |
|  |  |  |  |
| ICD 9 codes |  |  |  |
| dxvalue9 | ICD code | ICD description | |
| Sexual abuse | 995.83 | adult sexual abuse | |
|  | 995.53 | child abuse | |
|  |  |  |  |
| ICD 10 codes |  |  |  |
| dxvalue10 | ICD code | ICD description | |
| Sexual Abuse | T74.2 | sexual abuse confirmed | |
|  | T74.21 | adult sexual abuse confirmed | |
|  | T74.21XA | initial encounter | |
|  | T74.21XD | subsequent encounter | |
|  | T74.21XS | sequela |  |
|  | T74.22 | child sexual abuse confirmed | |
|  | T74.22XA | initial encounter | |
|  | T74.22XD | subsequent encounter | |
|  | T74.22XS | sequela |  |
|  | T74.5 | forced sexual exploitation confirmed | |
|  | T74.51 | adult forced sexual exploitation | |
|  | T74.51XA | initial encounter | |
|  | T74.51XD | subsequent encounter | |
|  | T74.51XS | sequela |  |
|  | T74.52 | child sexual exploitation confirmed | |
|  | T74.52XA | initial encounter | |
|  | T74.52XD | subsequent encounter | |
|  | T74.52XS | sequela |  |
|  | T76.2 | sexual abuse suspected | |
|  | T76.21 | adult sexual abuse suspected | |
|  | T76.21XA | initial encounter | |
|  | T76.21XD | subsequent encounter | |
|  | T76.21XS | sequela |  |
|  | T76.22 | child sexual abuse suspected | |
|  | T76.22XA | initial encounter | |
|  | T76.22XD | subsequent encounter | |
|  | T76.22XS | sequela |  |

Supplementary Material
